# Diffusion tensor imaging along the perivascular space and disease progression in people with multiple sclerosis: a 5-year longitudinal MRI study

**DOI:** 10.1093/braincomms/fcag002

**Published:** 2026-01-08

**Authors:** Ashley Tranquille, Robert Zivadinov, Bianca Weinstock-Guttman, Svetlana P Eckert, David Hojnacki, Michael G Dwyer, Niels Bergsland

**Affiliations:** Department of Neurology, Buffalo Neuroimaging Analysis Center, Jacobs School of Medicine and Biomedical Sciences, University at Buffalo, State University of New York, Buffalo, NY 14203, USA; Department of Neurology, Buffalo Neuroimaging Analysis Center, Jacobs School of Medicine and Biomedical Sciences, University at Buffalo, State University of New York, Buffalo, NY 14203, USA; Center for Biomedical Imaging at the Clinical Translational Science Institute, University at Buffalo, State University of New York, Buffalo, NY 14203, USA; Department of Neurology, State University of New York, Buffalo, NY 14203, USA; Department of Neurology, State University of New York, Buffalo, NY 14203, USA; Department of Neurology, State University of New York, Buffalo, NY 14203, USA; Department of Neurology, Buffalo Neuroimaging Analysis Center, Jacobs School of Medicine and Biomedical Sciences, University at Buffalo, State University of New York, Buffalo, NY 14203, USA; Department of Neurology, Buffalo Neuroimaging Analysis Center, Jacobs School of Medicine and Biomedical Sciences, University at Buffalo, State University of New York, Buffalo, NY 14203, USA

**Keywords:** multiple sclerosis, DTI-ALPS, MRI disease progression

## Abstract

Diffusion Tensor Image Analysis ALong the Perivascular Space (DTI-ALPS) was originally proposed to quantify glymphatic functioning. Although a direct interpretation is now questioned, cross-sectional studies show associations with disability in people with multiple sclerosis (pwMS). Regardless, serial DTI-ALPS studies are largely lacking in MS. In a longitudinal study, we investigated DTI-ALPS with respect to confirmed disability progression (CDP) and progression independent of relapse activity (PIRA) in people with relapsing-remitting MS (pwRRMS) and progressive MS (pwPMS). This study included 72 pwRRMS, 27 pwPMS, and 23 healthy controls (HC) imaged with 3T MRI and again after 5 years. The DTI-ALPS index was calculated using an automated pipeline using template-defined regions of interest (ROIs) in the superior longitudinal fasciculus and superior corona radiata. Areas corresponding to T2 hyperintensities were removed to avoid the influence of overt pathology. CDP and PIRA were assessed after 5 years and in 64 pwRRMS/17 pwPMS after 10 years. Comparisons between those with and without follow-up CDP or PIRA were assessed using analysis of covariance and repeated including normal appearing white matter (NAWM) mean diffusivity (MD) as an additional covariate. Multivariable binary logistic regression was used to explore whether DTI-ALPS offers independent value beyond general disease burden. Although significantly lower in pwMS compared with HCs (1.347 ± 0.178 versus 1.437 ± 0.132, *P* = 0.034, partial *η*^2^ = 0.021), the difference was no longer so after controlling for NAWM MD (*P* = 0.094, partial *η*^2^ = 0.024). DTI-ALPS decreases over 5 years were similar between HC and pwMS (*P* = 0.188, partial *η*^2^ = 0.021). In pwRRMS, baseline DTI-ALPS was lower in those who developed CDP or PIRA at 5- and 10 years of follow-up (all *P* ≤ 0.019, partial *η*^2^ > 0.080, except for PIRA at 5 years, *P* = 0.051, partial *η*^2^ = 0.055). When controlling for NAWM MD, results were in line with original findings. Baseline T2-LV was the only retained imaging predictor of CDP and PIRA over 5 years while only baseline DTI-ALPS was selected for in 10 year models. No associations were found in the pwPMS group. Changes in DTI-ALPS over 5 years did not relate to CDP nor PIRA in neither group. In conclusion, although DTI-ALPS values were not significantly different compared with HCs after considering NAWM MD, decreased baseline DTI-ALPS is associated with disability progression in pwRRMS. The lack of associations in pwPMS suggests that DTI-ALPS may be less informative with more advanced disease.

## Teaser

Tranquille *et al*. report findings from an MRI study using the Diffusion Tensor Image Analysis ALong the Perivascular Space technique. The authors show associations with disability progression in people with relapsing-remitting multiple sclerosis, but not those with a progressive phenotype, over 5 and 10 years of clinical follow-up.

## Graphical Abstract

**Graphical Abstract:**
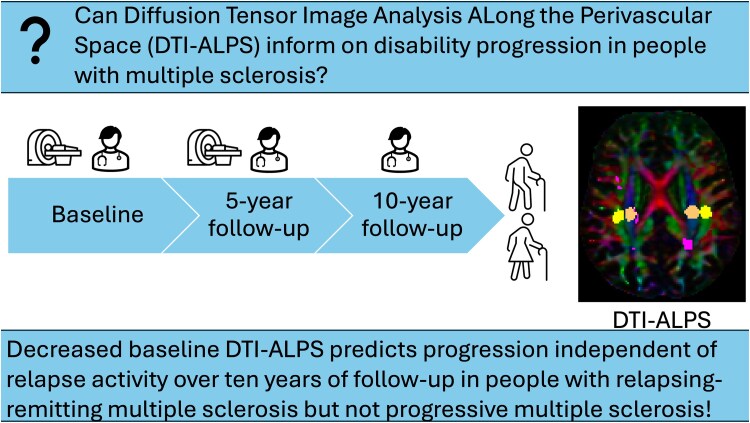

## Introduction

The Diffusion Tensor Image Analysis ALong the Perivascular Space (DTI-ALPS) method was initially developed as an *in vivo* method to assess impairment of the glymphatic system,^1^ which has been proposed to be involved in the clearance of metabolic waste from the brain.^2^ The technique garnered considerable attention in the literature due to its ease of application, including in retrospective datasets where a diffusion acquisition suitable for standard DTI analysis is available. However, it has become increasingly evident that the technique is an imperfect measure of glymphatic function at best^3^ or having nothing to do with it whatsoever.^4^ Ultimately, as acknowledged by the original authors of the method, the technique quantifies Brownian motion of water molecules in the radial direction at the lateral ventricular body level^1,5^ and cannot be directly interpreted as a measure of glymphatic functioning. Regardless, decreased DTI-ALPS values have been linked to worse neuroimaging and clinical outcomes in a number of neurodegenerative disorders.^6,7^ This is also the case for multiple sclerosis (MS), where it has been associated with greater lesion volumes, more advanced tissue atrophy,^8,9^ and increased disability.^8^

Accumulation of disability in pwMS is associated with both relapse-associated worsening and progression independent of relapse activity (PIRA). Although both are important, it is the latter that is thought to contribute to long-term disability.^10,11^ This is supported by the fact that most people with MS (pwMS) progress in their disability despite use of modern therapies capable of minimizing acute inflammatory attacks. While PIRA is strongly associated with neurodegeneration,^12^ tissue loss is the last phase of a complex interplay between ongoing pathological processes. The identification of earlier markers associated with disability progression may potentially help to identify pwMS at greater risk of progression with the aim of intervening with more aggressive treatment strategies. In this regard, cross-sectional associations between DTI-ALPS and disability have been reported.^8,9^ However, it remains largely unknown whether DTI-ALPS can inform on future disability progression as well as how DTI-ALPS changes over time in pwMS.

With this background, we hypothesized that DTI-ALPS at baseline would be associated with disability progression over 5- and 10-years of follow-up. We also hypothesized that pwMS would experience greater decreases in DTI-ALPS index over 5 years of follow-up compared with healthy controls (HC) and that such decline would relate to disability progression. Given that DTI-ALPS is not a specific marker of underlying pathology in neurodegenerative diseases,^3^ we also explored the effect of controlling for mean diffusivity (MD) of the normal appearing white matter (NAWM) in our analyses. This was done to assess whether the DTI-ALPS index provides additional explanatory power beyond that which can already be obtained from standard DTI of the NAWM, which has previously been shown to be informative of tissue injury^13^ and clinical disability in MS.^14^

## Materials and methods

### Study population

Study participants were from a prior longitudinal study investigating cardiovascular, environmental, and genetic factors in multiple sclerosis (CEG-MS) that began in 2009.^15^ The CEG-MS study's inclusion criteria included participants who were between 18 and 75 years of age and met the 2010 revision of the McDonald criteria for the diagnosis of MS. CEG-MS exclusion criteria included contraindications for MRI examination, pregnancy or nursing, and clinical relapse or administration of intravenous corticosteroid therapy within 30 days of the MRI examination. HCs, group matched for age and sex, were included on the basis of having no prior history of neurological or psychiatric disorders and normal neurological examination. Additional inclusion criteria for the present study included the availability of MRI and clinical assessment baseline MRI scan within 30 days of each other. All eligible participants with a diffusion-weighted imaging acquisition suitable for DTI-ALPS analysis were included. Participants were excluded from the current study if did not meet all of the criteria mentioned above or artefacts on baseline MRI prevented analysis.

All study participants returned for follow-up after an average of 5.4 ± 0.6 years, while a subset returned again after an average of 9.6 ± 1.6 years, hereafter referred to as year 5 and year 10 visits, respectively. Both follow-up visits included imaging and clinical assessments. Year 10 imaging was not analyzed due to differences in imaging protocols that might impact DTI-ALPS measures.

Approval for the CEG-MS study was obtained from the Institutional Review Board of the University at Buffalo, and all participants provided written informed consent in accordance with the Declaration of Helsinki.

### Clinical assessment

Physical disability for all pwMS was quantified by a neurologist specialized in MS or a Neurostatus-licensed investigator using the Expanded Disability Status Scale (EDSS)^16^ at all study visits. Confirmed disability progression (CDP) was defined as a definite EDSS increase between a baseline and follow-up visit, at least 6 months apart, sustained for at least one additional follow-up visit at least 6 months after the initial follow-up.^17^ A definite increases was defined as having an EDSS change of ≥ 1.5 if baseline EDSS was < 1.0, an EDSS change of ≥ 1.0 if baseline EDSS was 1.0–5.5, and an EDSS change of ≥ 0.5 if baseline EDSS was ≥ 5.5, as previously reported.^17,18^ PIRA was defined as having met the above criteria for CDP in the absence of a relapse as per the Lublin *et al*.^19^ definition. Clinical data from routine follow-up clinical visits that occurred between study visits were used for assessing PIRA. While PIRA is thought to play a larger role in contributing to long-term disability accumulation, we also included CDP, which includes relapse-associated worsening (RAW) as well, in order to provide a more comprehensive understanding of disease progression. Over the follow-up, a given study participant study could potentially have had both RAW and PIRA. Due to the limited number of individuals with RAW, we were unable to analyze these events on their own.

### Image acquisition

All image acquisitions were performed on the same 3T GE scanner (Signa Excite HD 12.0; GE, Milwaukee, WI, USA) with an eight-channel head and neck coil. The imaging protocol included an axial 3D T1-weighted (T1w) inversion recovery fast spoiled-gradient echo (TE = 2.8 ms; TI = 900 ms; TR = 5.9 ms; flip angle, 10°; isotropic 1 mm resolution) acquisition along with the following axially acquired 2D acquisitions with 1 × 1 × 3 mm^3^ voxel slices without gap: spin-echo T1w imaging (TE = 16 ms; TR = 600 ms); T2-weighted (T2w) fluid-attenuated inversion recovery (FLAIR) [TE = 120 ms; inversion time (TI) = 2100 ms; TR = 8500 ms; flip angle = 90°; echo-train length, 24]; dual fast spin-echo proton density weighted/T2w (TE1 = 9 ms; TE2 = 98 ms; TR = 5300 ms; echo-train length = 14); 2D diffusion imaging with 2.5 × 2.5 × 3mm^3^ voxels (reconstructed on the scanner to 1.25 × 1.25 × 3mm^3^) with 15 diffusion-weighted directions at *b* = 800 s/mm^2^ and one *b* = 0 s/mm^2^.

### Image processing

T2-FLAIR lesions were quantified using a semi-automated contouring technique, as previously reported.^20^ Normalized brain volume (NBV), normalized grey matter volume (NGMV), and normalized white matter volume (NWMV) were calculated using SIENAX with a lesion filled 3D T1 image.^21,22^ A NAWM mask was obtained by masking out lesions from the SIENAX white matter segmentation.

**Figure 1:**
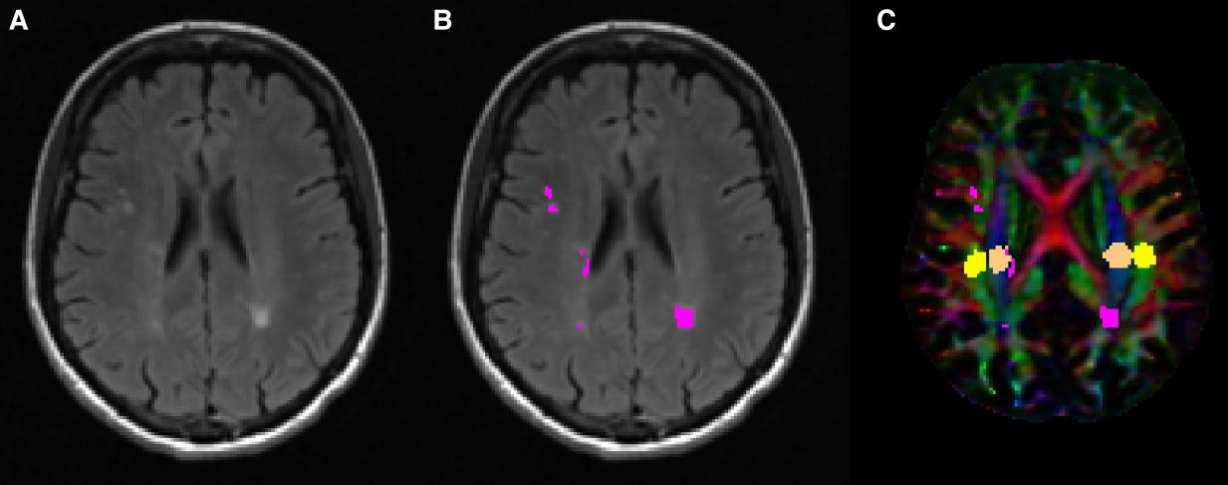
**Representative images from a person with multiple sclerosis.** (**A**) shows the T2-weighted fluid-attenuated inversion recovery (FLAIR) image from a representative person with multiple sclerosis. (**B**) shows the same T2-weighted FLAIR image with segmented T2 lesions overlaid in pink. (**C**) shows the color-coded primary vector modulated by the corresponding fractional anisotropy image. Lesions are again shown in pink while the SLF (association fibre) is shown in yellow and the corona radiata (projection fibre) is shown in orange.

DTI-ALPS index was calculated automatically from the diffusion-weighted data using a modified version of a previously published pipeline.^23^ Specifically, Synb0-DisCo^24^ was used to generate a synthetic *b* = 0 image without susceptibility-induced geometric distortions for subsequent processing with FSL’s topup.^25^ The data was then fed into FSL’s eddy^26^ for head motion and eddy current-induced geometric distortion correct after which DTI parameters were calculated using FSL’s dtifit. In addition, the lesion filled 3D T1 image was non-linearly registered to MNI 1 mm space using Advanced Normalization Tools (ANTs)^27^ and the distortion-corrected *b* = 0 image was rigidly aligned to the 3D T1 image. Afterwards, the *b* = 0 registration was concatenated with the non-linear warp and then inverted to bring MNI-defined regions of interest (ROIs) of the superior corona radiata (SCR) and superior longitudinal fasciculus (SLF) into diffusion space. As previously described, the ROIs were automatically defined as spheres with 5 mm diameter in the areas of bilateral SCR and SLF.^23^ T2 lesion masks were also brought into diffusion space for subsequent masking with the aforementioned ROIs to calculate DTI-ALPS measures only within NAWM. ROI placement was visually checked for all cases. As we did not have an a priori hypothesis regarding laterality differences, we computed a single DTI-ALPS measure reflecting the average value of the left and right ROIs. See Fig. 1 for a representative example of the ROI placement. In addition, the NAWM mask was used to calculate average NAWM MD.

### Statistical analyses

Statistical analyses were completed using IBM SPSS Statistics for Windows (version 29.0. Armonk, NY: IBM Corporation). Demographic characteristics were compared between HC and pwMS. The pwMS group was further broken down and assessed independently as pwRRMS and pwPMS subgroups. PwMS were also split into groups based on their CDP and PIRA status at both follow-up visits. For primary analyses, ANCOVA, controlling for age and sex, was used to assess group differences in imaging outcomes with effect sizes quantified using partial *η*^2^. Secondary analyses included NAWM MD as an additional covariate to control for overall tissue injury, as has been suggested recently by the authors of the DTI-ALPS method.^3^ Finally, exploratory analyses utilized multivariable logistic regression models to predict disability progression and assess whether the DTI-ALPS technique offers independent value beyond general disease burden. Specifically, the first block forced age, sex, and disease duration as predictors while DTI-ALPS, NAWM MD, T2-LV, and NBV were selected using forward stepwise procedures.

*P*-values < 0.05 were considered statistically significant.

## Results

### Demographics and clinical characteristics

The initial cohort included 99 pwMS and 35 HCs with available DTI-ALPS imaging data at baseline. Of these, 65 pwMS had 5-year follow-up imaging data with the same imaging protocol and were included in longitudinal DTI-ALPS analyses. In addition, 6 HCs were excluded due to having T2 lesion burden beyond that which was expected for their age and considered potentially pathological. At baseline, there were 72 people with relapsing-remitting MS (pwRRMS) and 27 people with progressive MS (pwPMS). After 5-years of follow-up, 33.3% of pwMS had CDP, corresponding to 30.3% having had PIRA and 5.1% having had RAW. Over the 5-year follow-up period, 2 pwMS (2.02%) had experienced both PIRA and RAW. Disability outcomes were assessed at 10 years in 64 pwRRMS and 17 pwPMS who returned for 10-year follow-up, with 44.4% of pwMS having had CDP, corresponding to 40.7% having had PIRA and 9.9% having had RAW. Of these, 5 pwMS (6.17%) had experienced both PIRA and RAW. Demographic and clinical characteristics of the 99 pwMS and 29 HCs are shown in Table 1 with full details, including disease modifying treatment usage are provided in Supplementary Table 1.

**Table 1:** Demographic and clinical characteristics of the study participants

	HC (*n* = 23)	pwMS (*n* = 99)	*P*-value	pwRRMS (*n* = 72)	pwPMS (*n* = 27)	*P*-value
Age in years, mean (SD)	41.9 (15.2)	45.4 (11.6)	0.223	42.3 (11.3)	53.3 (8.0)	<0.001
Sex, F (%)	14 (60.9%)	76 (76.8%)	0.120	52 (72.2%)	24 (88.9%)	0.073
Disease duration in years, mean (SD)	-	14.5 (11.0)	-	11.0 (8.56)	24.0 (11.16)	<0.001
Baseline EDSS, Median (IQR)	-	3.0 (1.5–5.0)	-	2.0 (1.5–3.0)	6.0 (4.5–6.5)	<0.001
5-year EDSS, median (IQR)	-	3.5 (2.0–6.0)	-	2.5 (1.5–3.9)	6.5 (5.0–7.0)	<0.001
10-year EDSS, median (IQR)	-	3.5 (2.0–6.0)	-	2.5 (1.5–4.0)	6.5 (5.0–7.5)	<0.001
Time to 5-year follow-up, mean (SD)	5.51 (0.48)	5.37 (0.60)	0.274	5.29 (0.65)	5.59 (0.39)	0.037
Time to 10-year Follow-up, mean (SD)	-	9.47 (1.51) (*n* = 60)		9.55 (1.50) (*n* = 45)	9.23 (1.57) (*n* = 15)	0.484

EDSS, Expanded Disability Status Scale; F, female; HC, healthy control; *N*, number; pwMS, people with multiple sclerosis; pwPMS, people with progressive multiple sclerosis; pwRRMS, people with relapsing-remitting multiple sclerosis; SD, standard deviation.

*P*-values derived from chi-square test, Student's *t*-test, and Mann-Whitney U-test, as appropriate.

### Baseline MRI imaging characteristics

Baseline imaging characteristics across groups are shown in Table 2. The DTI-ALPS index was significantly lower in pwMS compared with HCs (1.347 ± 0.178 versus 1.437 ± 0.132, *P* = 0.034, partial *η*^2^ = 0.038), although not after controlling for NAWM MD (*P* = 0.094, partial *η*^2^ = 0.024). PwPMS presented with numerically lower DTI-ALPS scores compared with pwRRMS but results were no longer significant after controlling for age and sex (1.27 ± 0.20 versus 1.37 ± 0.16, *P* = 0.169, partial *η*^2^ = 0.020, with *P* = 0.238, partial *η*^2^ = 0.015 after controlling for NAWM MD). DTI-ALPS was significantly correlated with NAWM MD in pwRRMS (*r* = −0.216, *P* = 0.039) but not in pwPMS (−0.014, *P* = 0.944) nor HCs (−0.165, *P* = 0.451).

**Table 2:** MRI imaging characteristics at baseline of HC and patients with multiple sclerosis

	HC (*n* = 23)	pwMS (*n* = 99)	*P*-value	Partial $\eta^{2}$	pwRRMS (*n* = 72)	pwPMS (*n* = 27)	*P*-value	Partial $\eta^{2}$
DTI-ALPS (unitless)	1.437 (0.132)	1.347 (0.178)	**0.034**	0.038	1.374 (0.162)	1.275 (0.201)	0.169	0.020
NAWM MD (×1000 mm^2^/s)	0.797 (0.035)	0.819 (0.041)	**0.038**	0.036	0.815 (0.043)	0.828 (0.036)	0.308	0.011
T2 lesion volume (mL)	0.015 (0.039)	16.5 (21.8)	**<0.001**	0.081	13.1 (18.8)	25.5 (26.7)	**<0.001**	0.120
Normalized brain volume (mL)	1577.7 (106.5)	1492.4 (109.2)	**<0.001**	0.078	1522.4 (97.4)	1412.5 (99.2)	**<0**.**001**	0.145
Normalized white matter volume (mL)	805.3 (47.8)	752.9 (55.1)	**<0.001**	0.113	765.1 (51.3)	720.2 (52.5)	**<0.001**	0.165
Normalized grey matter volume (mL)	772.4 (65.8)	739.5 (67.7)	**0.086**	0.025	757.3 (60.8)	692.2 (63.2)	**0**.**009**	0.077

DTI-ALPS, Diffusion Tensor Image Analysis ALong the Perivascular Space; HC, healthy control; mL, millilitre; NAWM MD, normal appearing white matter mean diffusivity; pwMS, people with multiple sclerosis; pwPMS, people with progressive multiple sclerosis; pwRRMS, people with relapsing-remitting multiple sclerosis.

Cells are shown in terms of mean (standard deviation).

*P*-values and partial $\eta^{2}$ effect sizes are derived from ANCOVA models corrected for age and sex. *P*-values < 0.05 are shown in bold.

### Associations between baseline DTI-ALPS and disability progression

**Figure 2:**
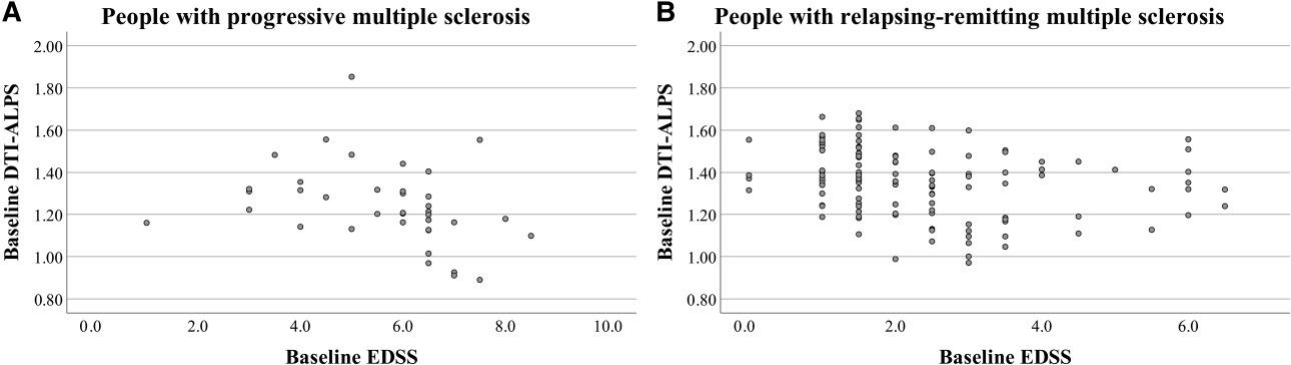
**Lower baseline DTI-ALPS values are associated with increased clinical disability.** Scatterplots showing the association between EDSS and DTI-ALPS values at baseline. Each point represents an individual with either progressive multiple sclerosis (**A**, *N* = 27) or relapsing-remitting multiple sclerosis (**B**, *N* = 72).

Baseline DTI-ALPS was more strongly associated with baseline EDSS scores in pwPMS compared with pwRRMS (Spearman's *ρ* = −0.544, *P* = 0.011 versus *ρ* = −0.300, *P* = 0.060) (Fig. 2). Differences in the association were further amplified when controlling for NAWM MD (*ρ* = −0.564, *P* = 0.010 versus *ρ* = −0.226, *P* = 0.167). Table 3 shows baseline the DTI-ALPS index compared between pwMS groups, and split by CDP and PIRA status at follow-up visits. Overall, baseline DTI-ALPS was associated with disability progression in pwRRMS, but not in pwPMS. Specifically, baseline DTI-ALPS was significantly lower in pwRRMS with CDP at year 5 compared with those without (1.306 ± 0.167 versus 1.404 ± 0.152, partial *η*^2^ = 0.080 *P* = 0.017). Weaker findings were evidenced when including NAWM MD as an additional covariate (partial *η*^2^ = 0.056, *P* = 0.050). Numerically lower baseline DTI-ALPS was seen in pwRRMS with PIRA at year 5 compared with those without (1.310 ± 0.173 versus 1.397 ± 0.153, *P* = 0.051, partial *η*^2^ = 0.055), with a weaker effect after controlling for NAWM MD (partial *η*^2^ = 0.028, *P* = 0.169). Results at year 10 were in line with those seen at year 5. Specifically, baseline DTI-ALPS was significantly lower in pwRRMS with CDP compared with those without (1.309 ± 0.163 versus 1.409 ± 0.150, partial *η*^2^ = 0.097, *P* = 0.014), even after controlling for NAWM MD (partial *η*^2^ = 0.074, *P* = 0.034). Similarly, DTI-ALPS was significantly lower in pwRRMS with PIRA versus without (1.309 ± 0.163 versus 1.409 ± 0.150, partial *η*^2^ = 0.088, *P* = 0.019), albeit with a weaker effect after including NAWM MD as an additional covariate (partial *η*^2^ = 0.060, *P* = 0.060).

**Table 3:** Baseline DTI-ALPS associations with disability progression

		Year 5				Year 10			
		No	Yes	*P*-value	Partial $\eta^{2}$	No	Yes	*P*-value	Partial $\eta^{2}$
CDP	pwRRMS	1.404 ± 0.152 (*n* = 50)	1.306 ± 0.167 (*n* = 22)	**0.017**	0.080	1.409 ± 0.150 (*n* = 40)	1.309 ± 0.163 (*n* = 24)	**0**.**014**	0.097
	pwPMS	1.226 ± 0.153 (*n* = 16)	1.345 ± 0.247 (*n* = 11)	0.276	0.051	1.189 ± 0.180 (*n* = 5)	1.330 ± 0.241 (*n* = 12)	0.717	0.010
PIRA	pwRRMS	1.397 ± 0.153 (*n* = 53)	1.310 ± 0.173 (*n* = 19)	0.051	0.055	1.405 ± 0.153 (*n* = 43)	1.304 ± 0.158 (*n* = 21)	**0**.**019**	0.088
	pwPMS	1.226 ± 0.153 (*n* = 16)	1.345 ± 0.247 (*n* = 11)	0.276	0.051	1.189 ± 0.180 (*n* = 5)	1.330 ± 0.241 (*n* = 12)	0.717	0.010

CDP, confirmed disability progression; DTI-ALPS, Diffusion Tensor Image Analysis ALong the Perivascular Space; PIRA, progression independent of relapse activity; pwPMS, people with progressive multiple sclerosis; pwRRMS, people with relapsing-remitting multiple sclerosis.

All cells are shown in terms of mean (standard deviation). DTI-ALPS is a unitless measure.

*P*-values and partial $\eta^{2}$ effect sizes are derived from ANCOVA models corrected for age and sex. Significant *P*-values < 0.05 are shown in bold.

In binary logistic regression analyses of disease progression over 5 years in pwRRMS, increased baseline T2-LV was the only variable retained for predicting CDP (odds ratio (OR) = 1.907, 95% confidence interval (CI) [1.012, 3.593], Wald = 3.990, *P* = 0.046, Nagelkerke *R*^2^ = 0.161) and PIRA (OR = 2.303, 95% CI [1.153, 4.600], Wald = 5.590, *P* = 0.018, Nagelkerke *R*^2^ = 0.203). In analysis of disease progression over 10 years in pwRRMS, lower baseline DTI-ALPS was the only variable retained for predicting CDP (OR = 0.259, 95% CI [0.092, 0.732], Wald = 6.492, *P* = 0.011, Nagelkerke *R*^2^ = 0.337) and PIRA (OR = 0.255, 95% CI [0.088, 0.739], Wald = 6.339, *P* = 0.012, Nagelkerke *R*^2^ = 0.307).

No imaging measures were retained in any of the models investigating disability progression in pwPMS.

Re-running the statistical analysis after excluding those who had both PIRA and RAW did not affect the findings other than slightly larger *P*-values and marginally wider confidence intervals (Results not shown).

### Changes in DTI-ALPS over 5 years

Change in DTI-ALPS over 5 years was not significantly different between HCs and pwMS (−0.103 ± 0.119 versus −0.069 ± 0.108, *P* = 0.188, partial *η*^2^ = 0.021) nor between any of the subgroups (data not shown). Table 4 shows changes in DTI-ALPS over 5 years with respect to disability progression measures after 5 and 10 years of follow-up. Change in DTI-ALPS index was not significantly associated with either CDP or PIRA at either follow-up visit, regardless of whether or not NAWM MS was included as a covariate.

**Table 4:** Longitudinal 5- and 10-year CDP and PIRA association with change in DTI-ALPS

		Year 5				Year 10			
		No	Yes	*P*-value	Partial $\eta^{2}$	No	Yes	*P*-value	Partial $\eta^{2}$
CDP	pwRRMS	−0.081 ± 0.114 (*n* = 30)	−0.090 ± 0.063 (*n* = 12)	0.827	0.001	−0.115 ± 0.111 (*n* = 19)	−0.061 ± 0.56 (*n* = 9)	0.163	0.080
	pwPMS	−0.029 ± 0.106 (*n* = 15)	−0.049 ± 0.139 (*n* = 8)	0.638	0.012	−0.075 ± 0.105 (*n* = 5)	−0.010 ± 0.106 (*n* = 8)	0.226	0.158
PIRA	pwRRMS	−0.081 ± 0.111 (*n* = 32)	−0.092 ± 0.068 (*n* = 10)	0.776	0.002	−0.112 ± 0.106 (*n* = 21)	−0.055 ± 0.061 (*n* = 7)	0.169	0.077
	pwPMS	−0.029 ± 0.106 (*n* = 15)	−0.049 ± 0.139 (*n* = 8)	0.638	0.012	−0.075 ± 0.105 (*n* = 5)	−0.010 ± 0.106 (*n* = 8)	0.226	0.158

CDP, confirmed disability progression; DTI-ALPS, Diffusion Tensor Image Analysis ALong the Perivascular Space; PIRA, progression independent of relapse activity; pwPMS, people with progressive multiple sclerosis; pwRRMS, people with relapsing-remitting multiple sclerosis.

All cells are shown in terms of mean (SD). DTI-ALPS is a unitless measure. *P*-values and partial $\eta^{2}$ effect sizes are derived from ANCOVA models corrected for age and sex.

## Discussion

Despite the substantially decreased enthusiasm for DTI-ALPS as an *in vivo* measure of glymphatic functioning, results from our study suggest that the technique can be useful in the study of MS. We found that baseline DTI-ALPS values, derived from ROIs in the NAWM, were lower in pwMS compared with HCs, albeit significance becoming lost when controlling for mean NAWM MD in the brain.

We also assessed the association between DTI-ALPS with respect to disability, both at baseline and over the follow-up. In an earlier cross-sectional study, it was suggested that the DTI-ALPS index is especially decreased in the progressive stages of the disease.^8^ Our results showing stronger associations between baseline DTI-ALPS and EDSS measures in the pwPMS group in agreement with those earlier findings. However, our findings diverge when considering CDP and PIRA over five and ten years of follow-up. Specifically, we found that baseline DTI-ALPS measures were associated with future disease progression, even after controlling for NAWM MD, in the pwRRMS, but not in pwPMS. These findings suggest that the DTI-ALPS measure may be more clinically relevant in terms of determining further disability accumulation over the mid-term follow-up earlier in the disease. This notion is further supported by the multivariable binary logistic regression analyses. Specifically, with baseline DTI-ALPS, NAWM MD, T2-LV, and NBV measures as potential predictors of progression, only DTI-ALPS was retained for CDP and PIRA over 10 years of follow-up in pwRRMS. Although changes in DTI-ALPS over 5 years of follow-up were not related to progression over 5 and 10 years of clinical follow-up, pre-existing tissue injury may play a role in determining which patients go on to accumulate disability. If true, this would suggest that the diffusivity changes reflected by DTI-ALPS occur early in the disease and subsequently slow down over time.

Contrary to our initial hypothesis, we did not detect significant differences in 5-year DTI-ALPS change between the HCs and pwMS groups. As of now, there are very few longitudinal DTI-ALPS studies in the literature that have included HCs, rendering it difficult to draw firm conclusions regarding how this measure changes over time in healthy individuals. One study that investigated the longitudinal pattern of DTI-ALPS measures in a sample of HC found that it decreased at an annualized rate of ∼−0.093,^7^ which is substantially greater in magnitude than what we found in our HC groups (−0.103 over 5 years, or −0.019 on an annualized basis). The reasons for this discrepancy are unknown at this time but may be due to differences in method of ROI placement and/or potential inclusion of areas of T2 lesions (i.e. white matter hyperintensities) in ROIs analyzed in the aforementioned study. We specifically limited our ROIs to the NAWM as white matter hyperintensities have altered diffusivity properties,^28^ the inclusion of which would potentially violate some of the basic assumptions of the DTI-ALPS method. Regardless, additional longitudinal studies are needed to clarify how DTI-ALPS evolve in pwMS compared with HCs.

Our study is not without its limitations. First, our diffusion imaging protocol was characterized by a limited number of diffusion-weighted directions, although there is not any reason to suspect that this would have biased the results one way or the other. Second, the pwRRMS group was substantially larger than the pwPMS group, which could have prevented us from identifying associations with progression outcomes in the latter. However, this concern is somewhat mitigated by considering effect size measures, which support the notion that the DTI-ALPS measure is more relevant in the earlier disease stages with respect to disability progression. Nevertheless, studies with larger sample sizes are needed; especially when assessing relationships between changes in DTI-ALPS and disability accumulation, as the pwPMS group without CDP or PIRA over 10 years had numerically greater decreases in DTI-ALPS. Third, the DTI-ALPS index is likely affected by disparate biological processes, especially in the context of a neurodegenerative disease like MS.^3^ We attempted to address this by including NAWM MD as an additional covariate in our statistical analyses, which had the effect of weakening our results to some degree. Moreover, while we focused on the DTI-ALPS index calculated from the standard ROIs used for this method, it might be possible that DTI-ALPS measures obtained from other areas in the brain may have similar explanatory power. As previously mentioned, considering the DTI-ALPS measure as a direct measure of glymphatic dysfunction is no longer tenable. Studies focusing specifically on glymphatic functioning should utilize methods that are likely more specific (e.g. contrast-enhanced MRI T1 mapping,^29^ multiple echo time arterial spin labelling,^30^ intravoxel incoherent motion MRI^31^). Finally, the pwMS included in our study were mostly on first line therapies, limiting the potential to assess the impact of higher efficacy treatments on the associations between DTI-ALPS and disability progression.

Despite the decreased enthusiasm for DTI-ALPS as a measure of glymphatic functioning, its use as another tool to investigate neurodegeneration should not be discarded. Results from our study suggest that baseline DTI-ALPS is associated with disability progression over the mid-term follow-up in pwRRMS. Nevertheless, more work is required to validate our findings before it can be considered as a prognostic marker. Future studies should compare DTI-ALPS to more establish imaging correlates of disability progression, including brain atrophy, diffuse NAWM injury, and chronic active inflammation.

## Supplementary Material

fcag002_Supplementary_Data

## Data Availability

Data is available from the corresponding author (N.B.) upon reasonable request. Raw images cannot be made available due to lack of data sharing consent from study participants.
