# Supplementary material for: Diffusion tensor imaging along the perivascular space and disease progression in people with multiple sclerosis: a 5-year longitudinal MRI study
Source: Brain Commun. 2026 Jan 8;8(1):fcag002. doi: 10.1093/braincomms/fcag002 (PMC12820730; doi:10.1093/braincomms/fcag002)
Supplement: fcag002_Supplementary_Data [file fcag002_supplementary_data.docx]

**Supplementary Table 1. Full demographic and clinical characteristics of the study participants.**

|  | HC (n=23) | pwMS (n=99) | p-value | pwRRMS (n=72) | pwPMS (n=27) | p- value |
| --- | --- | --- | --- | --- | --- | --- |
| Age in years, mean (SD) | 41.9 (15.2) | 45.4 (11.6) | .223 | 42.3 (11.3) | 53.3 (8.0) | < 0.001 |
| Sex, F (%) | 14 (60.9%) | 76 (76.8%) | .120 | 52 (72.2%) | 24 (88.9%) | 0.073 |
| Disease duration in years, mean (SD) | - | 14.5 (11.0) | - | 11.0 (8.56) | 24.0 (11.16) | < 0.001 |
| Baseline EDSS, Median (IQR) | - | 3.0 (1.5-5.0) | - | 2.0 (1.5-3.0) | 6.0 (4.5-6.5) | < 0.001 |
| DMT use at baseline, n (%) | - |  | - | - | - | 0.098 |
| Interferon-β | - | 49  (49.5) | - | 38 (52.8) | 11 (40.7) |  |
| Glatiramer acetate | - | 18 (18.2) | - | 10 (13.9) | 8 (29.6) |  |
| Fingolimod |  | 1 (1.0) |  | 1 (1.4) | 0 (0.0) |  |
| Natalizumab | - | 15 (15.2) | - | 12 (16.7) | 3 (11.1) |  |
| Off-label^#^ | - | 4 (4.0) | - | 1 (1.4) | 3 (11.1) |  |
| None | - | 12 (12.1) | - | 10 (13.9) | 2 (7.4) |  |
| 5-year EDSS, median (IQR) | - | 3.5 (2.0-6.0) | - | 2.5 (1.5-3.9) | 6.5 (5.0-7.0) | < 0.001 |
| 10-year EDSS, median (IQR) | - | 3.5 (2.0-6.0) | - | 2.5 (1.5-4.0) | 6.5 (5.0-7.5) | < 0.001 |
| Time to 5-year follow-up, mean (SD) | 5.51 (0.48) | 5.37 (0.60) | 0.274 | 5.29 (0.65) | 5.59 (0.39) | 0.037 |
| Time to 10-year Follow-up, mean (SD) | - | 9.47 (1.51)  (n= 60) |  | 9.55 (1.50)  (n=45) | 9.23 (1.57)  (n= 15) | 0.484 |
| CDP at 5-year follow-up, Yes (%) | - | 33 (33.3%) | - | 22  (30.6%) | 11  (40.7%) | 0.349 |
| PIRA at 5-year follow-up, Yes (%) | - | 30 (30.3%) | - | 19  (26.4%) | 11  (40.7%) | 0.220 |
| RAW at 5-year follow-up, Yes (%) | - | 5 (5.1%) | - | 4 (5.6%) | 1 (3.7%) | 1.000 |
| CDP at 10-year follow-up, Yes (%)^ | - | 36 (44.4%) | - | 24 (37.5%) | 12 (70.6%) | 0.026 |
| PIRA at 10-year follow-up, Yes (%)^ | - | 33 (42.0%) | - | 21 (32.8%) | 12 (70.6%) | 0.011 |
| RAW at 5-year follow-up, Yes (%)^ | - | 8 (9.9%) | - | 6 (9.4%) | 2 (11.8%) | 0.672 |

**Legend**: HC – healthy control; pwMS – people with multiple sclerosis; pwRRMS – people with relapsing-remitting multiple sclerosis; pwPMS – people with progressive multiple sclerosis; n – number; SD – standard deviation; F – female; EDSS – Expanded Disability Status Scale; DMT – disease modifying therapy; IQR – interquartile range; SD – standard deviation; CDP – confirmed disability progression; PIRA – progression independent of relapse activity; RAW – relapse-associated worsening

P-values derived from chi-square test, Student's t-test, and Mann-Whitney U test, as appropriate.

^ - 64 pwRRMS and 17 pwPMS had 10-year follow-up data available; # - Off-label treatments included intravenous immunoglobulin (n=1), methotrexate (n=1), mitoxantrone (n=1), and mycophenolate mofetil (n=1).
